# A Mycobacteriophage-Based Vaccine Platform: SARS-CoV-2 Antigen Expression and Display

**DOI:** 10.3390/microorganisms9122414

**Published:** 2021-11-23

**Authors:** Krista G. Freeman, Katherine S. Wetzel, Yu Zhang, Kira M. Zack, Deborah Jacobs-Sera, Sara M. Walters, Dominique J. Barbeau, Anita K. McElroy, John V. Williams, Graham F. Hatfull

**Affiliations:** 1 Department of Biological Sciences, University of Pittsburgh, Pittsburgh, PA 15260, USA; kgf10@pitt.edu (K.G.F.); ksw34@pitt.edu (K.S.W.); KMZ30@pitt.edu (K.M.Z.); djs@pitt.edu (D.J.-S.); 2 UPMC Children’s Hospital of Pittsburgh, Pittsburgh, PA 15260, USA; yuz113@pitt.edu (Y.Z.); SMM293@pitt.edu (S.M.W.); MCELROYA@pitt.edu (A.K.M.); jvw@chp.edu (J.V.W.); 3 Center for Vaccine Research, Department of Pediatrics, University of Pittsburgh School of Medicine, Pittsburgh, PA 15260, USA; DJB176@pitt.edu

**Keywords:** phage display vaccine, mycobacteriophage, SARS-CoV-2

## Abstract

The explosion of SARS-CoV-2 infections in 2020 prompted a flurry of activity in vaccine development and exploration of various vaccine platforms, some well-established and some new. Phage-based vaccines were described previously, and we explored the possibility of using mycobacteriophages as a platform for displaying antigens of SARS-CoV-2 or other infectious agents. The potential advantages of using mycobacteriophages are that a large and diverse variety of them have been described and genomically characterized, engineering tools are available, and there is the capacity to display up to 700 antigen copies on a single particle approximately 100 nm in size. The phage body may itself be a good adjuvant, and the phages can be propagated easily, cheaply, and to high purity. Furthermore, the recent use of these phages therapeutically, including by intravenous administration, suggests an excellent safety profile, although efficacy can be restricted by neutralizing antibodies. We describe here the potent immunogenicity of mycobacteriophage Bxb1, and Bxb1 recombinants displaying SARS-CoV-2 Spike protein antigens.

## 1. Introduction

Bacteriophage-based vaccine systems have been explored using a variety of specific platforms, but none are yet in routine clinical use in humans [1]. The COVID-19 pandemic illustrates the need for flexibility in vaccine development, and the potential roles of platforms such as those based on mRNA, adenovirus systems, and nanoparticles have been explored [2]. Continued exploration of phage-based systems offers the potential for new low cost, high production vaccines, and the global pandemic of SARS-CoV-2 provides a context for such development.

Bacteriophage vaccine platforms include the single-stranded DNA filamentous phage M13 platform used primarily in phage display technology [3,4,5,6] and several double-stranded DNA (dsDNA) tailed phage systems such as *Escherichia coli* phages T4 [7,8,9], T7 [10,11], and lambda [12,13,14,15]. Typically, the phage capsid of these dsDNA-tailed phages is used to display a foreign protein or part of a protein, most commonly through non-covalent linkages [5]. Several such recombinant phage systems have been shown to stimulate strong immune responses, which can be protective through neutralization of the infectious agent carrying the antigen [9,11,15]. Notably, two recent publications [16,17] describe innovative phage-based vaccine approaches for SARS-CoV-2, including phage display of antigens, delivery of phage genomes encoding antigens within mammalian expression cassettes, and encapsulation of antigenic proteins within the phage capsid. The evident advantages of such systems are the simplicity and relatively low cost of production, the adjuvantal nature of the phages themselves [1], and the strong safety profile of bacteriophages demonstrated during therapeutic use [18,19]. However, there are potential limitations with the need to prevent lipopolysaccharide (LPS) contamination from the *E. coli* host, growth and stability of the phages, and potential loss of antigen during purification [20]. Other phage systems are thus worth exploring, and a large number of phages of other bacterial hosts have been described.

The largest collection of phages known to infect a single common bacterial host are those of *Mycobacterium smegmatis* (referred to as mycobacteriophages). Over 10,000 have been isolated of which over 2000 genomes have been sequenced and annotated [21,22]. These are part of a larger collection of over 18,000 phages that infect various bacteria within the phylum *Actinobacteria*, over 3000 of which are genomically characterized [22]. Their genomes are highly diverse but can be grouped according to overall sequence relationships into ‘clusters’ (Cluster A, B, C, etc.), some of which can be readily divided into ‘subclusters’ (Subcluster A1, A2, A3, etc.) [22]. Some of the phages have no close relatives and these are referred to as ‘singletons’. The genetic diversity is such that even within these closely related clusters and subclusters there is substantial sequence variation and there are few examples of the same phage being independently isolated [22].

SARS-CoV-2 is a new coronavirus that has been intensely studied since its discovery in late 2019. Vaccine development efforts have focused on its spike (S) protein, which decorates the lipid membrane and makes direct contact with the human angiotensin-converting enzyme 2 (ACE2) receptor [23]. Vaccines developed by Moderna [24,25] and Pfizer [26] use mRNAs coding for the S protein, although with mutations that promote expression of the pre-fusion S protein conformation. The Oxford/AstraZeneca vaccine [27] delivers S protein DNA in an adenovirus vector, the Inovio vaccine [28] gives S DNA by electroporation, and the Novavax [29] platform uses nanoparticles decorated with S protein. Structural studies and comparisons with SARS-CoV-1 show that a 197-amino acid Receptor Binding Domain (RBD) is required for interaction with human ACE2, and that its 72-residue Receptor Binding Motif (RBM) makes direct contacts with ACE2 and is a target for neutralizing antibodies [30].

Mycobacteriophages have been used therapeutically to treat *Mycobacterium* infections and have strong safety profiles following intravenous administration [18]. Many grow well to high titer, are readily purified, and are stably maintained. Nonetheless, in immunocompetent patients extended intravenous application can elicit potent neutralizing antibody responses [31]. Understanding mycobacteriophage immunogenicity will therefore advance both their therapeutic use and their potential in vaccine development. No mycobacteriophage-based vaccines have been reported previously, although *Mycobacterium*-based vaccine platforms have been described, typically using mycobacteriophage-derived genetic tools [32,33]. Here we explore the concept of COVID-19 vaccine development using mycobacteriophages to display SARS-CoV-2 RBM segments. Using phage phiTM45—a lytic derivative of parent Bxb1 [34]—we constructed several recombinants displaying peptides at high density on the phage capsid, and showed that they can stimulate IgG-mediated antibody responses in mice to the displayed SARS-CoV-2 RBM epitopes as well as to the phage itself. Mouse sera showed poor neutralization against SARS-CoV-2, presumably due to conformational issues of antigen presentation, but despite this, the mycobacteriophage platform has attractive features as a general vaccine development tool.

## 2. Materials and Methods

### 2.1. Bacterial Strains

All recombineering, phage isolation, and phage amplification used *Mycobacterium smegmatis* mc^2^ 155 [35]. In liquid, *M. smegmatis* mc^2^ 155 was cultured at 37 °C in Middlebrook 7H9 (Difco, Detroit, MI, USA) supplemented with 0.2% glucose and 0.05% Tween 80. Cultures were grown to saturation over 2–4 days. On solid media, *M. smegmatis* mc^2^ 155 was grown on Middlebrook 7H10 (Difco, Detroit, MI, USA) supplemented with 0.5% glycerol and 0.2% glucose. When used to amplify phage, these media were supplemented with 1 mM CaCl_2_.

### 2.2. Construction of Vaccine Candidates

All phages discussed here are derivatives of phiTM45, a spontaneously lytic mutant isolated from phage Bxb1 [36]. Vaccine candidates (Table 1) are classified as either Bacteriophages Displaying Antigens of SARS-CoV-2 (BaDAS) or DNA Encoded and Displayed Antigens of SARS-CoV-2 (DEaDAS). BaDAS-1, BaDAS-3, BaDAS-4, and DEaDAS-1 were constructed using the CRISPY-BRED technique, as previously described [37]. In brief, phenol-chloroform extraction was used to purify DNA from high titer lysates of phiTM45 and BaDAS-1. The phage DNA and a substrate containing the desired insertion sequence (codon optimized for expression in mycobacteria, in the case of the BaDAS engineering) flanked on both sides by 250 bp of homologous DNA were co-electroporated into cells containing the recombineering plasmid pJV138 [38]. Cells were recovered at 37 °C for 3.5–4.5 h in 7H9 supplemented with 10% ADC and 1mM CaCl_2_, then mixed with *M. smegmatis* mc^2^ 155: pIRL53 carrying a designed sgRNA, and plated on lawns of 7H10/ADC/KAN/CaCl_2_/100ng/mL anhydrotetracycline (ATc). After ~24 h of growth, plaques were screened for the desired mutation via PCR and sequenced as described previously [39]. BaDAS-5, BaDAS-6, and BaDAS-7 were constructed by subjecting BaDAS-1, BaDAS-3, and BaDAS-4 to selection on a lawn of *M. smegmatis* mc^2^ 155: pIRL53 carrying a sgRNA targeting a DNA sequence in the 3’ end of gene *19*. Escapees were screened for deletions with plaque PCR. pcDNA3-SARS-CoV-2-S-RBD-8his, used in the generation of DEaDAS-1, was a gift from Erik Procko (Addgene plasmid # 145145; http://n2t.net/addgene:145145; RRID:Addgene_145145)

### 2.3. Phage Amplification

The phages were amplified on solid media following a modified, large-scale, plate-based protocol. In brief, 7H10 supplemented with 0.5% glycerol, 0.2% glucose, and 1mM CaCl_2_ was poured into autoclaved cafeteria style serving trays and allowed to solidify. For each tray, 1–5 × 10^5^ pfu of phage was added to 20 mL of saturated *M. smegmatis* mc^2^ 155 culture and allowed to adsorb for 15 min at room temperature. 200 mL of Middlebrook Top Agar (MBTA; Middlebrook 7H9, 3.5 g/L BactoAgar, 1 mM CaCl_2_) was added to the infection and the mixture was poured onto the prepared solid media. The trays were incubated at 30 °C for ~40 h, then the top agar was harvested and centrifuged at 8000 rpm for 15 min. The phage-rich supernatant was recovered and treated with DNase I (Sigma Aldrich, St. Louis Missouri, USA) for 30 min at room temperature, then filtered through a 0.2 μm membrane and centrifuged at 100k× *g* for 1 h to pellet the phage. The phage pellet was resuspended in phage buffer (10 mM Tris, pH 7.5, 10 mM MgSO_4_, 68 mM NaCl) and CsCl was added to 850 g/L. This was centrifuged at 38 k rpm for 16 h to further purify via CsCl gradient, then the resulting phage band was extracted through side puncture and stored at 4 °C for future use. Before mouse trials, phage was dialyzed in a 15 kD cutoff dialysis bag at 4 °C against a 1000× volume of phage buffer supplemented with 1 mM CaCl_2_. Dialysis in phage buffer proceeded for at least 6 h before an exchange to fresh buffer, then again for at least 6 h or overnight. Finally, the sample was dialyzed once overnight against a 1000× volume of PBS (Sigma Aldrich, St. Louis, MO, USA) supplemented with 1 mM CaCl_2_ and 1 mM MgCl_2_. Samples were stored at 4 °C.

### 2.4. Vaccine Stability Test

The thermostability of phage-based vaccine candidates were assessed by first measuring the initial titer of CsCl-banded phage samples (by performing 10× serial dilutions into PBS supplemented with 1 mM CaCl_2_ and 1 mM MgCl_2_, adsorbing 10 µL of each dilution to 300 µL *M. smegmatis* mc^2^ 155, mixing with top agar and plating on Middlebrook 7H10 agar plates), then transferring 50 µL of the same samples to a plastic screw-cap tube and storing in a refrigerator (nominal temperature 4 °C). At the designated timepoints, the titers of the samples were again measured.

### 2.5. Mouse Studies

Adult C57BL/6J mice were immunized in groups of 3–5 with BaDAS and DEaDAS vaccine candidates (see Table 2 for details of each study). For this study, where the primary interest is to assess the ability of vaccine candidates to induce S protein specific antibodies, relative to the control vaccinated groups (PBS, phiTM45, RBD + LPS, and S protein + LPS), a small sample size of 3–5 in each group is sufficient. Negative controls were done by immunizing mice with either PBS (mock) or wild type phiTM45 (parent phage, no SARS-CoV-2 antigen display). Subunit vaccine were administered as experimental positive controls by immunizing mice with either 20 µg SARS-CoV-2 Spike recombinant S1 protein + 5 µg LPS or 2 µg RBD + 2.5 µg LPS. Recombinant S1 and RBD proteins were both obtained from GenScript (Piscataway Township, Piscataway, NJ, USA, catalog numbers Z03501 and Z03479, respectively). Each bolus was injected intraperitoneally in 100 µL volume. Serum was collected by submandibular bleeding at indicated times after initial inoculation (Table 2). Most animals received a boost of similar size 2–3 weeks after the initial inoculation, with some receiving a third dose after 4 weeks. All mice were maintained in specific pathogen free conditions in accordance with the Institutional Animal Care and Use Committee of University of Pittsburgh. The University of Pittsburgh’s Institutional Animal Care and Use Committee has reviewed and approved the research proposal referenced above, approval code: Protocol #: 21028897, approval date: 23 February 2021.

### 2.6. ELISA

Enzyme-linked immunosorbent assays (ELISAs) were performed as previously described [31], except the wells were coated with either 5 × 10^8^ pfu phiTM45 or 100 ng of SARS-CoV-2 S1 protein (Z03501, GenScript, Piscataway Township, New Jersey, USA) in coating buffer. Control wells were incubated with pure coating buffer (no antigen). Briefly, coated wells were incubated at 4 °C overnight, blocked with 300 μL PBST + 5% BSA for at 4 °C overnight, filled with 100 µL of heat inactivated mouse serum (serially diluted in blocking buffer) and incubated at 4 °C overnight. The sera were decanted, then wells were incubated with 100 µL of preadsorbed, HRP-conjugated Goat Anti-Mouse IgG H&L (ab97040, abcam, Cambridge, UK), diluted 1: 50,000 into PBST, for 1 h in the dark at room temperature. Finally, plates were quantified by adding 100 µL of TMB substrate (T0440, Sigma Aldrich, St. Louis, MO, USA) to each well, developing for 8 min, adding 100 µL of 2N H_2_ SO_4_ to stop the reaction, then measuring the absorbance at 450 nm (signal) and at 570 nm (background). The background-subtracted values were plotted against the dilution and fit with a fixed baseline logistic curve using OriginLab software version 2021b 9.8.5.212 (Northampton, MA, USA). For each serum tested, the endpoint titer was determined by calculating the ratio of the logistic curve fits for the SARS-CoV-2 Spike S1 protein response to background response (wells coated with pure coating buffer, no antigen). For responsive animals, the greatest dilution at which this ratio was greater than 4 defined the endpoint dilution. Reciprocal serum endpoint titers were calculated as log_10_ (1/endpoint dilution).

### 2.7. Western Blot

Western blots were performed as previously described [31]. Briefly, denatured phage proteins and recombinant SARS-CoV-2 proteins were separated on 4–20% SDS-PAGE gradient gels, then transferred to PVDF membrane using either wet transfer (Towbin transfer buffer) or semi-dry transfer (discontinuous Tris/CAPS transfer buffer). The membrane was blocked overnight in TBS + 3% milk, incubated overnight in diluted mouse serum (typically a 1:1000 dilution in TBST + 3% milk), incubated for one hour with secondary antibody (Goat Anti-Mouse IgG H&L (HRP) preadsorbed; ab97040, Abcam, United Kingdom) diluted 1:40,000 in TBST + 3% milk, then developed with SuperSignal WestPico PLUS Chemiluminescent Substrate (ThermoFisher, Waltham, MA, USA and imaged with an Amersham Imager 600 (GE Healthcare, Chicago, IL, USA).

### 2.8. Phage Neutralization Assay

To test for potential neutralization of phage particles by serum antibodies, 1 µL of experimental serum (from mice immunized with either PBS, BaDAS-1, or BaDAS-5) was incubated with 100 µL of phage (at a titer of 1 × 10^9^ pfu/mL) at room temperature. After 0, 1, 2, 4, 22, and 50 h a sample was removed and the titer of active phage was measured (by performing 10× serial dilutions into PBS, adsorbing to *M. smegmatis* mc^2^ 155, and plating on Middlebrook 7H10 agar plates).

### 2.9. Focus Reduction Neutralization Test (FRNT)

Neutralization tests were performed as previously described [40]. Sera from a non-human primate previously vaccinated against SARS-CoV-2 and known to neutralize virus in this assay was included as a positive control. Raw foci numbers are reported. All work with live SARS-CoV-2 (clinical isolate from UPMC Hospital in June 2020) was performed at Biosafety Level 3 in the University of Pittsburgh Regional Biocontainment Laboratory.

## 3. Results

### 3.1. Mycobacteriophage phiTM45 as a Vaccine Platform Candidate

All of the mycobacteriophages described to date are dsDNA-tailed phages with genomes ranging from 40–160 kbp. Most have isometric capsids with icosahedral symmetry, although there is considerable sequence variation among the morphogenic genes [22]. The capsids typically are constructed from pentameric and hexameric capsomers arranged with quasi-symmetry, and there are several examples of chainmail-like interlinking of the capsomers [36]. The tails are 130–350 nm long and composed of an abundant tail tube protein, a central tape measure protein, and an additional 6–10 minor tail proteins, including the receptor binding proteins.

**Figure 1:**
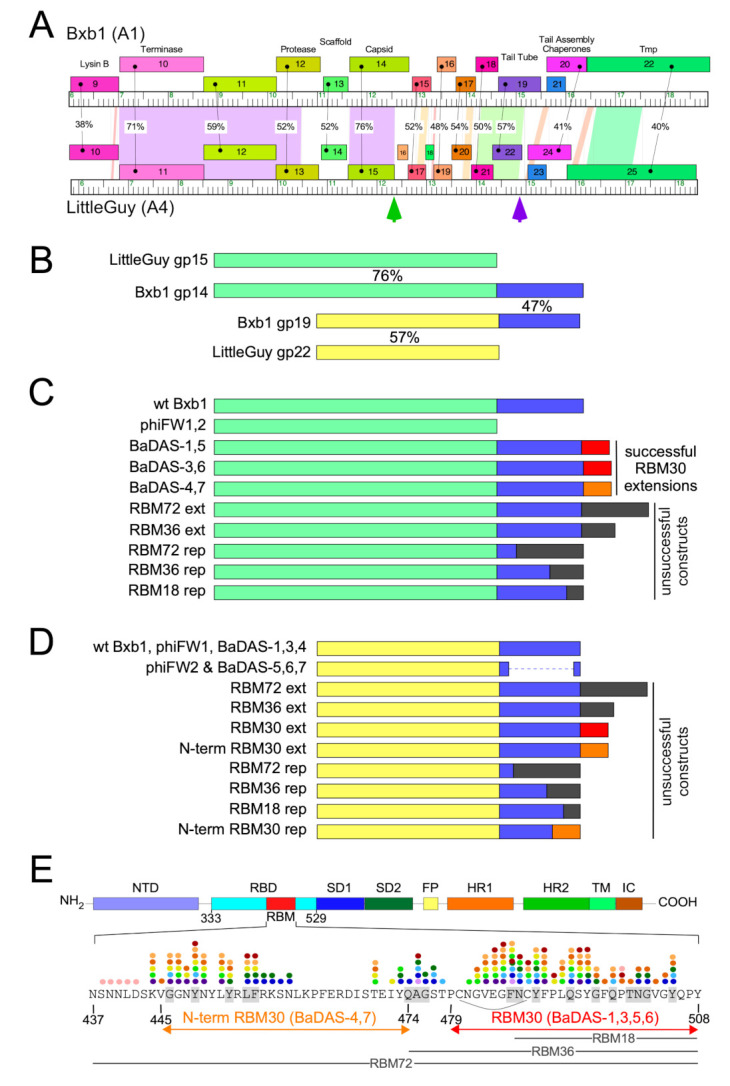
Design of Bacteriophage Displaying Antigens of SARS-CoV-2 (BaDAS) recombinant phages. (**A**) Alignment of phage Bxb1 (Subcluster A1) and LittleGuy (Cluster A4) virion structural genes. Genomes are represented as horizontal tracks with the predicted rightwards-transcribed genes shown as colored boxes above each genome. Gene names are shown in each box and genes are colored to indicate similarity. Spectrum colored shading between genomes shows pairwise BLASTN nucleotide alignment (violet strong similarity, red weakest similarity above threshold of BLASTN E-value of 10^−4^); amino acid identities (%) between genes are also shown. Green and purple vertical arrows indicate the Bxb1 capsid and tail tube C-terminal extensions. (**B**) Alignment of Bxb1 and LittleGuy capsid and tail tube proteins; shared capsid and tail tube protein segments are colored pale green and yellow, respectively, and the Bxb1 gp14 and gp19 extensions are shown in blue. Percent amino acid identities (%) are shown. (**C**) Schematic representations of Bxb1 recombinant phages. phiFW1 and phiFW2 lack the gp14 C-terminal extension, and BaDAS-1–BaDAS-7 have C-terminal SARS-CoV-2 RBM additions; red (RBM30) and orange (N-term RBM30) C-terminal additions correspond to the SARS-CoV-2 Spike protein regions in panel **E**. (**D**) Schematic representation of Bxb1 gp19 recombinant phages, colored as above. (**E**) SARS-CoV-2 Spike protein domain organization. Domains are depicted as described previously [23], with the Receptor Binding Domain (RBM) shown in red, and its sequence [46] shown below. The RBM segments displayed in BaDAS constructs (N-term RBM30 and RBM30, denoted with orange and red arrows, respectively) span residues binding to ACE2 [23,47,48] (gray highlighted residues) or various neutralizing antibodies, denoted with colored dots (see refs [49,50,51,52,53] for details). BaDAS-1, 3, 5, and 6 contain a disulfide bond stabilizing the RBM (indicated with an arc). The constructions containing regions RBM18, RBM36, and RBM72 (18, 36 and 72-residues, respectively, shown with gray labelled lines) were unsuccessful.

We previously noted an unusual feature of mycobacteriophage Bxb1 virions (Figure 1A), in which both the capsid and the tail tube subunits contain C-terminal regions (~90 amino acids) that are sequence-related to each other but absent from closely related phages (Figure 1B) [41]. Because these C-terminal ‘extensions’ are absent from related phages they are presumably not essential for virion assembly. The similarity of the capsid protein to the well-studied HK97 suggests that the capsid extensions are present on the outside of the shell [42,43]. The specific role of these extensions is not known, but we note that this general relationship is seen with some other mycobacteriophages (e.g., Wildcat) where the extensions contain Ig-like motifs, as described for many other phages [44,45]. We reasoned that these protein segments could be replaced by antigens of choice, on either the capsid, tail tube, or both, and that dozens of different phages could be exploited in this way. A phage such as Bxb1 has a T = 7 symmetry, facilitating presentation of 415 copies of an antigen on a single capsid, and a tail of 130 nm length with ~270 copies of the tail tube protein [36]. We therefore explored whether these capsid and tail proteins could be exploited for antigen display.

### 3.2. SARS-CoV-2 S Protein Epitopes for Display

We chose to display segments of the SARS-CoV-2 S RBM because it is both a critical component in viral infection as well as a vulnerable target for vaccines (Figure 1E). Several residues within the RBM form hydrogen bonds with the N-terminal helix of the human ACE2 [23,47,48], the receptor for SARS-CoV-2 infection [54,55]. Furthermore, many neutralizing antibodies against SARS-CoV-2 isolated from convalescent patient sera [49,50,51,52,53] bind to epitope residues within the RBM. The RBM is therefore a suitable component of SARS-CoV-2 to target for vaccine development. The ACE2-binding residues and neutralizing antibody epitope residues of the RBM are shown in Figure 1E. The segments chosen for constructing phage-based vaccine candidates span these critical residues.

### 3.3. Construction of the BaDAS Series of Vaccine Candidates

To engineer phage genomes we used the previously described CRISPY-BRED strategy [37]. In this approach, phage genomic DNA and a synthetic DNA substrate are electroporated into a recombineering strain of *M. smegmatis* to yield a mixture of parental and mutant phage progeny. These are then plated onto a strain expressing Cas9 and a single guide RNA (sgRNA) targeting the parental but not the mutant phage. The CRISPR-counter selection is typically sufficiently efficient that phage survivors are either the desired recombinants or CRISPR escape mutants (CEMs) with mutations in the targeted protospacer or associated PAM site [37]. The approach facilitates efficient construction of the desired recombinants, but also strongly indicates if a desired recombinant is non-viable, as then only CRISPR escape mutants are recovered among the progeny.

**Figure 2:**
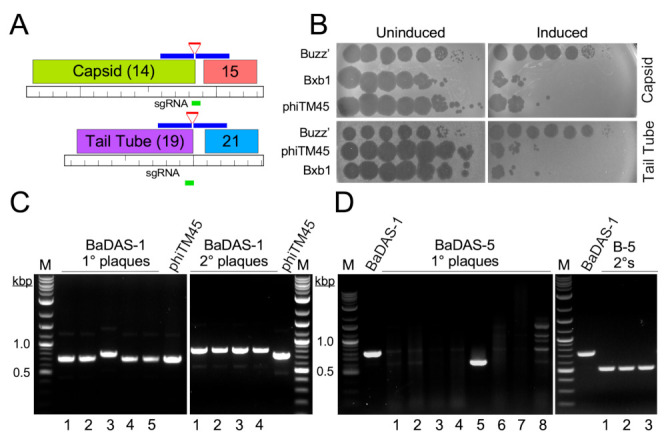
Construction of BaDAS recombinant phages. (**A**) Modification of the Bxb1 capsid and tail tube subunits. Locations of the sgRNA (green line) targeting either the capsid or tail tube subunit genes (gene *14* and *19*, top and bottom, respectively), and the synthetic DNA substrates (blue lines) carrying RBM sequences (red lines) are shown. (**B**) CRISPR-mediated counter selection. Plasmids with inducible expression of Cas9 and sgRNAs targeting the 3′ junctions of phage Bxb1 genes *14* or *19* (and its derivative phiTM45) were transformed into *M. smegmatis* mc^2^ 155. Cells were plated with or without ATc inducer (as indicated) and 10-fold serial dilutions of Bxb1, phiTM45, and a control phage, BuzzLyseyear, were spotted onto the lawns. sgRNA induction reduces Bxb1 (and phiTM45) plaquing by at least four orders of magnitude. Top panel, targeting capsid gene *14*; Bottom panel, targeting tail tube gene *19*. (**C**) Primary plaques (left panel) recovered from CRISPY-BRED engineering were screened by PCR using primers flanking gene *14*, and a candidate recombinant (candidate 3) was plaque purified and secondary plaques re-screened by PCR; all candidates are recombinant. (**D**) Primary plaques (left panel) recovered from CRISPR selection against wild type gene *19* were screened by PCR using primers flanking gene *19*, and a candidate recombinant (candidate 5) was plaque purified and secondary plaques re-screened by PCR (right panel); all candidates are recombinant.

Using the CRISPY-BRED strategy we attempted to construct recombinant phages displaying antigenic peptides of SARS-CoV-2 on the surface of phiTM45, a lytic derivative of phage Bxb1 (Figure 1C,D and Figure 2A; Table 1); these are referred to as Bacteriophages Displaying Antigens of SARS-CoV-2 (BaDAS) vaccine candidates. We first demonstrated that the native C-terminal extensions on the capsid and tail tube proteins are nonessential by truncating them to form phages phiFW1 and phiFW2, respectively (Figure 1C,D). The sgRNA counter selection against parent phages is efficient (Figure 2B) and the constructions were uncomplicated, confirming that both derivatives are viable. Next, we attempted to replace the native C-terminal extensions with SARS-CoV-2 peptides of varying lengths (RBM72rep, RBM36rep, and RBM18rep, containing 72, 36, and 18 amino acids, respectively). Surprisingly, we were unable to recover any of these, and only CEMs were isolated among all the phage derivatives recovered and tested. Thus, although these extensions are not required for viability, they cannot be readily replaced by non-phage sequences in the way we had hoped. Nonetheless, when characterizing the CEMs that were recovered during attempted capsid engineering, we identified a mutant with a single base pair deletion in the protospacer that introduces a frameshift mutation near the 3′ end of the capsid gene, resulting in addition of 30 amino acids encoded in the -1 reading frame at the capsid protein C-terminus. This suggested that although the native capsid protein extension is dispensable—yet irreplaceable—that it could be extended by at least 30 residues. Based on this observation we made three recombinants (BaDAS-1, 3, and 4; for clarity, BaDAS-2 is the same as BaDAS-3, but contained a SNP and was not analyzed further) in which 30 amino acid segments of the SARS-CoV-2 RBM are added to the capsid C-terminus (Figures 1C and 2A,C); BaDAS-1 and BaDAS-3 have the RBM C-terminal residues added with slightly different junction sequences, and BaDAS-4 has the RBM N-terminal residues (Figure 1E). Similar constructions were attempted for the tail protein but were not able to be made (Figure 1D). However, the use of CRISPR to select against the 3′ end of the tail tube gene in this attempt yielded CEMs with truncated extensions of the tail tube, giving phages BaDAS-5, 6, and 7 (Figures 1C and 2D). The resulting six variants of genetically modified phages display > 400 antigenic peptides on the capsid surface, without evident interference of virion assembly or structure (as supported by viability). All phage derivatives were confirmed by Sanger sequencing in the region of the mutation; BaDAS-1 was completely sequenced.

### 3.4. Construction of DEaDAS-1 Recombinant Vaccine Candidate

**Figure 3:**
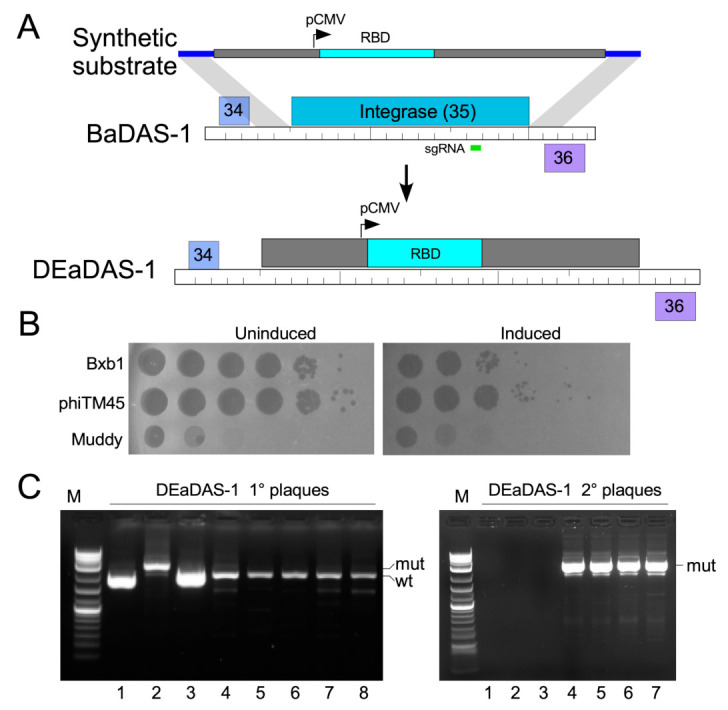
Construction of a DNA Encoded and Displayed Antigens of SARS-CoV-2 (DEaDAS) recombinant phage. (**A**) The integrase gene (blue) of phage phiTM45 was replaced with a mammalian expression cassette (gray box) containing the RBD of SARS-CoV2 (aqua) driven by a CMV promoter (pCMV). The location of the sgRNA target selecting against parent phiTM45 (green) is indicated. Regions of homology between the synthetic substrate and phiTM45 are indicated (blue line), and the structure of recombinant phage DEaDAS-1 is shown below. (**B**) sgRNA induction for selection against the integrase gene reduces Bxb1 and phiTM45 plaquing by at least two orders of magnitude. (**C**) Primary plaques (left panel) recovered from CRISPY-BRED engineering were screened by PCR, and a candidate recombinant (candidate 2) was plaque purified and secondary plaques (right panel) re-screened by PCR; all candidates are recombinant.

Bacteriophages can also be used to deliver DNA encoding targets for immune responses, as ‘DNA vaccines’ [56]. To explore whether these mycobacteriophages could be used similarly, we used the CRISPY-BRED approach to construct a recombinant carrying the S protein’s 197-residue RBD (Figure 1E) expressed from the cytomegalovirus (CMV) promoter (pCMV, Figure 3A). A 2495 bp mammalian expression cassette was amplified from pcDNA3-SARS-CoV-2-S-RBD-8his [57] (Addgene plasmid #145145, coordinates 138–2632) and ligated between two 250-bp gene blocks with homology to BaDAS-1, immediately upstream and downstream of gene *35* (Figure 3A). In a CRISPY-BRED experiment, the synthetic substrate and BaDAS-1 genomic DNA were co-electroporated into recombineering cells, and plaques were recovered on a sgRNA-expressing strain that counterselects against the parent phage (Figure 3B). Screening of primary plaques identified a desired recombinant (DNA Encoded and Displayed Antigens of SARS-CoV-2, DEaDAS) which was purified and confirmed by PCR and full genome sequencing (Figure 3C).

### 3.5. Characterization of Recombinant Phages

**Figure 4:**
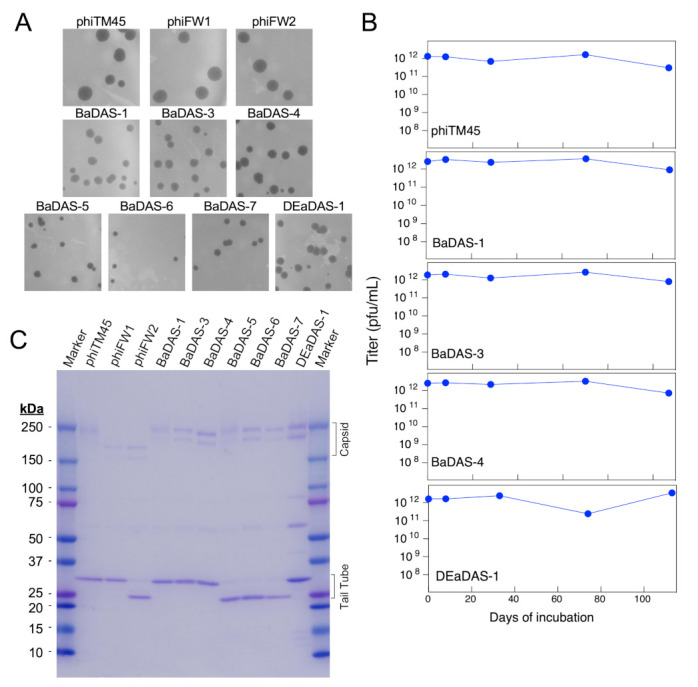
Characterization of BaDAS and DEaDAS recombinant phages. (**A**) phiTM45 and derivatives phiFW1 and phiFW2 form large plaques on *M. smegmatis*, whereas all other derivatives form somewhat smaller plaques. (**B**) Thermostability of high titer, highly purified preparations of phiTM45 and vaccine candidates were assessed by determining the titer after incubation at ~4 °C for different times. All phages were stable for ~100 days of simple refrigeration. (**C**) SDS-PAGE of virion proteins shows the impact of modifications of capsid and tail tube subunits.

Recombination to make the desired phages seemingly occurred with relatively low frequency compared to other constructions [37]. This may be due to a minor growth defect, which is indicated by the difference in plaque sizes between the wild type and recombinant phages. While phiTM45, phiFW1, and phiFW2 all produce large plaques, BaDAS-1,-3, and -4 and DEaDAS-1 each form smaller plaques and produce raw lysates of slightly lower titer compared to phiTM45 (Figure 4A). Similarly, BaDAS-5,-6, and -7 exhibit plaque sizes slightly smaller than their full-length tail counterparts (Figure 4A).

The choice of phage Bxb1 and its derivative phiTM45 as scaffolds for exploring vaccine construction was predicated in part because these can readily be grown to high titer and are stable when stored at 4 °C. To characterize the impact of virion modifications on phage stability, we incubated phiTM45, BaDAS-1, BaDAS-3, BaDAS-4, and DEaDAS-1 at ~ 4 °C and determined the phage titers at intervals up to at least 100 days (Figure 4B). All the phage preparations showed little or no reduction in titer over the 100-day period when stored cold at high (~1 × 10^12^ pfu/mL) concentration. These are encouraging parameters for potential vaccine development, especially in the context of a global pandemic where cold chain interruptions can spoil vaccines that must remain frozen.

Analysis of phage virion proteins confirmed the structures of the recombinant phages (Figure 4C). Phage phiTM45 has the expected profile with the most prominent bands being the tail tube subunit (predicted MW 30.1 kDa) and both pentameric and hexameric covalently interlinked capsid capsomers (predicted MW 209 and 250 kDa, respectively; Figure 4C) [36]. Phages phiFW2, BaDAS-5, BaDAS-6, and BaDAS-7 all have truncations of the tail tube gene and the major tail subunits are smaller as predicted, with molecular weights of ~23.5 kDa (Figure 4C). The truncation of the capsid subunit in phiFW1 and phiFW2 reduces the sizes of the capsid capsomers as expected (MW 164 and 197), and the small capsid additions in all BaDAS and DEaDAS phages have relatively little effect on capsid protein mobilities (Figure 4C).

### 3.6. Immune Responses in Mice

**Figure 5:**
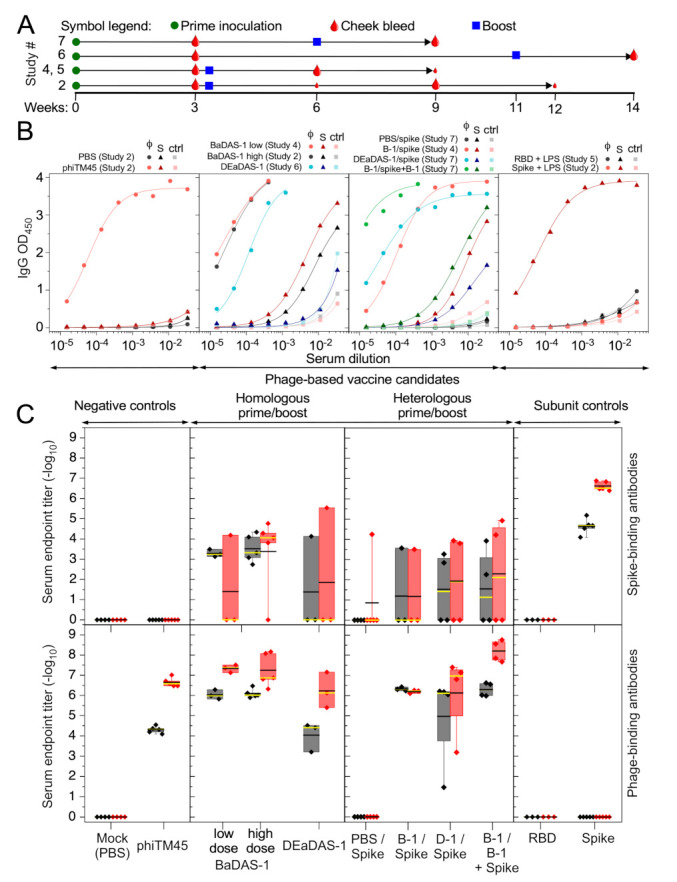
Immune responses of mice to effective phage-based vaccine candidates and controls. (**A**) Timelines for the subset of vaccination studies shown in this figure (timelines and additional study details are also included in Table 2). Each study begins with a prime inoculation (green circle) and was followed by a cheek bleed (red drop) at Week 3, but subsequent boost (blue square) and bleed schedules differed between studies as indicated. Large red drops indicate cheek bleeds for which corresponding data are shown in panels B and C, while ELISA data for cheek bleeds indicated with small red drops are not shown here. (**B**) IgG ELISA curves for phage-coated (φ), spike-coated (S), and uncoated (ctrl) wells are shown for a selection of responsive animals immunized with recombinant phages and control samples. The Study # for each dataset is indicated above each panel. BaDAS-1 and DEaDAS-1 (central two panels) both induce strong IgG responses to the phage moiety (e.g., phiTM45; circle symbols) and somewhat weaker response to Spike protein (triangle symbols) in these animals, in both homologous and heterologous regimens. Spike protein, but not RBD (both with LPS adjuvant), induce an IgG response (right panel) to Spike, but no response to the phage. Mock immunization with parent phage phiTM45 induces strong IgG responses to the phage but no response to Spike protein, while PBS immunization does not induce IgG responses to either (left panel). (**C**) Reciprocal serum endpoint titers of all inoculated animals for vaccine regimens represented in panel B. Top and bottom panels show end point titers against Spike protein and phage phiTM45, respectively. Titers for individual animals are shown as diamond symbols (with whiskers extending to the minima and maxima of each dataset) and box boundaries mark the first and third quartiles of the data. Black and yellow lines are the mean and median endpoint titer, respectively. Shaded to indicate responses after primary inoculation (gray) or the boost (red), with collection times as shown in panel A.

The immunogenicity of the BaDAS and DEaDAS vaccine candidates was determined by inoculation of C57BL/6J mice in a series of experiments testing for the effects of primary inoculation and either a homologous or heterologous boost (Figure 5A; Table 2). PBS, phage phiTM45, adjuvanted recombinant SARS-CoV-2 S protein, or adjuvanted RBD were included as controls, and IgG antibody responses were measured for both the S protein fragment and phiTM45 using an ELISA format (Figure 5B). Endpoint titers were deduced from ELISA titrations and are shown in Figure 5C.

First, these studies show that the phiTM45 phage itself is strongly immunogenic, and phage-specific antibodies are among the strongest reactions in all experiments (Figure 5B,C). Three weeks after the primary inoculation, IgG endpoint titers to the phage were typically stronger than 1:10,000 dilution and increased by 1–2 orders of magnitude following a boost that included phiTM45 phage or a phage recombinant. In homologous prime-boost combinations (both with BaDAS-1), similar reactions were observed to both low (7 × 10^10^ PFU) and high (2 × 10^11^ PFU) doses (Figure 5C). The DEaDAS-1 candidate was inoculated at a somewhat lower dose (5 × 10^9^ PFU) and gave somewhat weaker phage-specific antibody responses. The strongest phage-specific immune response was observed using BaDAS-1 as a primary inoculum and a boost of BaDAS-1 coupled with S protein (Figure 5C). No phage-specific reactions were seen using PBS, RBD or S protein controls (Figure 5C).

The BaDAS-1 candidate elicited S-specific immune responses in a homologous prime-boost experiment, but the reactions varied greatly among individual mice and included several animals in which little to no S-specific responses were observed; even the most robust reactions were weaker than those to the phage in the same animal (Figure 5B,C). These reactions were not substantially improved using a heterologous boost with recombinant S protein, or a combination of BaDAS-1 and S protein (Figure 5C). However, these reactions were in contrast with using adjuvanted RBD inoculations in which no mice mounted an S-specific reaction (Figure 5C). The lack of response observed to RBD + LPS may be due to low dosage, as other studies have shown robust responses to adjuvanted RBD in larger doses [58]. Adjuvanted S protein alone did elicit a strong and uniform response which was elevated by one order of magnitude following a homologous boost. The DEaDAS-1 inoculated animals responded similarly to the BaDAS-1 parent phage, but with more unresponsive animals, perhaps reflecting the use of the slightly lower inoculum size. Immune reactions to other BaDAS phages, at various dosages and in the absence or presence of adjuvant, were generally similar to those in Figure 5 and are shown in Figure S1. Western blots showing reactivity to purified RBD in sera from mice immunized with BADAS-1 are show in Figure S2.

### 3.7. Phage Capsid and Tail Tube Extensions Are Strongly Immunogenic

**Figure 6:**
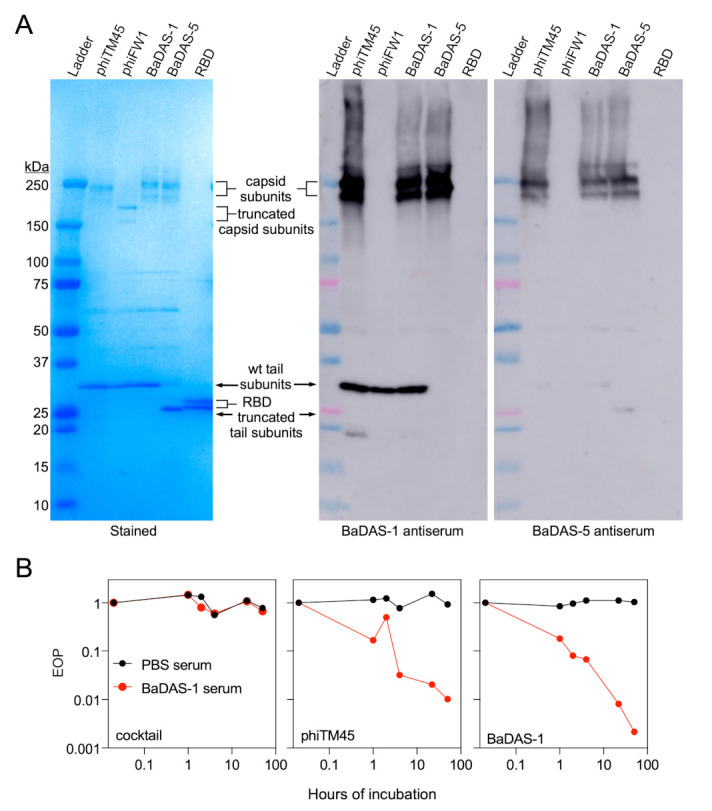
Native C-terminal extensions of Bxb1 capsid and tail proteins are immunodominant. (**A**) Coomassie-stained gel (left) and Western blots (right) of wild type phiTM45 and mutants phiFW1, BaDAS-1, and BaDAS-5, as well as recombinant SARS-CoV-2 RBD. When probed with serum from a mouse immunized with BaDAS-1, strong binding to wild type tail proteins is observed. In contrast, serum from a mouse immunized with the shaved-tail derivative, BaDAS-5, produces little to no binding to tail proteins. Robust binding to the capsid protein complexes of phiTM45, BaDAS-1 and BaDAS-5 is observed for both sera, while neither sera binds to the truncated capsid proteins of phiFW1. No binding to the SARS-CoV-2 RBD is observed from either sera, despite the presence of significant spike-binding antibodies as assayed by ELISA; this is due to the substantially stronger immune response to phage structural components compared to the display SARS-CoV-2 antigens. Western blots probing response to RBD only are shown in Figure S2. (**B**) A cocktail of unrelated phages, wild type phiTM45, and BaDAS-1 were incubated with sera from mice immunized with PBS or BaDAS-1 and, after the specified duration of incubation, titered. While the cocktail of unrelated phages were unaffected by this treatment, both phiTM45 and BaDAS-1 were substantially neutralized by the BaDAS-1 antiserum (but unaffected by the mock infection serum).

The strong (endpoint titers generally exceeding 1:1,000,000) immune reactions to the phage are quite striking, and we therefore explored these reactions further. Probing a Western blot of phiTM45 phage particles with serum from a BaDAS-1 immunized mouse showed strong reactivity to both the tail tube subunit, and the capsid capsomers (Figure 6A). Interestingly, the same serum recognized the tail tube subunit of phiFW1, but does not recognize its truncated capsid capsomers, suggesting that the antibody response is primarily recognizing the natural C-terminal extension on the capsid subunit (Figure 6A). Similarly, this serum recognizes the capsid capsomers of both BaDAS-1 and BaDAS-5, but only recognizes the tail tube protein of BaDAS-1; it does not recognize the tail tube subunit of BaDAS-5 from which the C-terminal extension has been removed (Figure 6A).

These observations indicate that the C-terminal capsid extension on phiTM45 is highly immunogenic. The tail tube subunit may also be highly immunogenic, but because the amino acid sequences of these are related to each other (47% identity, Figure 1A), it is possible that antibodies raised against the capsid extension cross-react with the tail tube extension. To examine this, we probed a similar Western blot with serum from a BaDAS-5 (which lacks the tail tube extension) inoculated mouse (Figure 6A). Antibodies in this serum recognized the capsid capsomers of phiTM45, BaDAS-1, and BaDAS-5 but not phiFW1 which lacks the capsid C-terminal extension. However, the tail tube subunit is not recognized for any phage suggesting that the C-terminal extensions on the capsid and on the tail tube are independently immunogenic, rather than cross-reacting to the same antibodies (Figure 6A). Further data supporting this conclusion are shown in Figure S3. We note that the ELISAs suggest that the BaDAS-1 immune response that is specific to the S protein component is stronger than that induced by BaDAS-5, suggesting that the tail tube extension is more likely to be immunostimulatory than acting as an immune distraction.

Although the function of the C-terminal extensions on the capsid and tail tube proteins is not known, their immunogenicity is intriguing. Because they are not essential for infection, antibody binding to them is not expected to strongly neutralize phage infection. To test this, we incubated lysates of phiTM45, BaDAS-1, or an unrelated phage with serum from a BaDAS-1 inoculated mouse for up to 50 h and measured the phage titer (Figure 6B). We observed some phage neutralization of phiTM45 and BaDAS-1, with a reduction of about two orders of magnitude over the first six hours, and greater but incomplete neutralization at the longer time points. It seems likely that this arises from a subset of antibodies that recognize the minor tail tip proteins that interact with the host receptor rather than those recognizing the capsid and tail tube. We note that in contrast, serum from a phage therapy patient had a robust phage-specific immune response that was capable of completely neutralizing (>10^6^ fold reduction in titer) the therapeutic phage in a two-hour incubation [31]. Thus, the neutralization of BaDAS-1 is relatively mild in light of the strong IgG anti-phage response (Figure 5B). Bxb1 has not been used therapeutically in humans and a direct comparison is not available.

### 3.8. Spike-Binding Antibodies Raised in Mice Do Not Neutralize SARS-CoV-2 In Vitro

**Figure 7:**
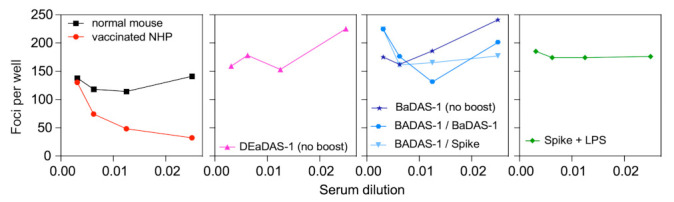
No in vitro neutralization of SARS-CoV-2 by immunized mouse sera. Focus reduction neutralization tests were performed with sera from mice immunized with controls and vaccine candidates. The left panel shows assay controls: the negative control (normal mouse serum) has 125–150 foci per well at all serum dilutions, while incubation with the positive control (vaccinated non-human primate serum) reduces the number of foci per well. No reduction in foci was observed after incubation with any of the experimental sera, including that from mice immunized with Spike + LPS (right panel). This lack of neutralization likely results from unproductive conformations of the Spike protein and displayed epitopes.

To assess the protective potential of the S-binding antibodies generated in response to BaDAS and DEaDAS vaccine candidates, we performed a Focus Reduction Neutralization Test (FRNT). Positive (vaccinated non-human primate serum) and negative (normal mouse serum) controls neutralized or did not, respectively, as expected (Figure 7, left panel) but no reduction in the number of foci per well was observed after incubation with any experimental sera (Figure 7 central panels). In fact, slight increases in the number of foci are observed in nearly every well treated with the experimental sera. Surprisingly, even serum from mice immunized with recombinant S protein with LPS showed no SARS-CoV-2 neutralization (Figure 7 right panel); this is likely a consequence of the post-fusion conformation adopted by the recombinant wild type S protein. Additional FRNT results are shown in Figure S4.

We note that antibody-dependent enhancement (ADE) of SARS-CoV-2 infection in vitro has been reported even for neutralizing antibodies isolated from convalescent plasma [59,60]. In the case of RBD-binding antibodies, ADE occurs when the Fc of the virus-bound antibody binds to cell surface Fc-gamma receptors [60]. This traffics the virus to the cell surface and promotes uptake via endocytosis. Notably, despite ADE of infection in vitro, the same antibodies protect from SARS-CoV-2 replication in mice and monkeys.

## 4. Discussion

Phage-based vaccines have several attributes making them attractive as platforms for protection against infections. These include their relatively low cost and ease of production, strong safety profile reflected in phage therapeutic use, and their potential adjuvantal properties. They can be engineered to both display immunogenic epitopes on their surface to deliver hundreds of immunogens on nanoscale particles and to simultaneously serve as DNA vaccine delivery vehicles. Here, we show that mycobacteriophages such as Bxb1 and its derivatives can be genetically manipulated, and we have advanced our understanding of the opportunities and limitations of peptide display on the capsid subunits. We have also demonstrated that recombinant phages can be constructed with the potential to act as DNA vaccine delivery systems.

The BaDAS and DEaDAS vaccine candidates were constructed in the year that COVID-19 pandemically spread across the globe and when a broad range of strategies in COVID-19 vaccine development was an urgent response. Thus, there was a strong motivation for the construction and evaluation of mycobacteriophage derivatives with the potential to protect against SARS-CoV-2 infection. Display of S protein 30-residue epitopes was achieved, and immune responses were observed in contrast with adjuvanted RBD protein, suggesting immunological benefits from phage display. However, there was surprisingly high variation in immune response to the displayed epitope among individual mice, unlike the uniform antibody response to the phage itself. We do not understand the basis of this observation. Moreover, even when antibody binding to the displayed peptide was observed, it did not confer neutralization of SARS-CoV-2 activity, most likely due to the inability of the peptide to fold into a suitable conformation to give neutralizing immune reactions. In contrast, we note that a T4-based phage display vaccine displaying full-length S protein trimers was effective in generating antibodies that both bind the S protein and neutralize SARS-CoV-2 infection [17]. It is plausible that epitopes from other pathogens, or longer peptides from SARS-CoV-2 S protein, would not suffer from this conformational limitation and further exploration of this vaccine platform is warranted.

The Bxb1-derived phages have some remarkable features. The natural C-terminal extensions on the capsid and tail tube proteins are not required for phage infection and replication, as predicted from comparative genomic analyses, and both can be removed. However, simple replacement of all or part of these extensions is not tolerated, at least for the limited number of constructions attempted here. Further studies are needed to explore these limitations and potentially to overcome them. Preliminary structural studies indicate that the capsid C-terminal domain is indeed external to the capsid shell but forms distinctly different assemblies at the pentameric and hexameric capsomers (KGF and GFH, manuscript in preparation). The inter-subunit packing of the C-terminal domain is much tighter in the hexameric capsomer than in the pentameric capsomer suggesting that it may be a hexamer-specific problem. We also were unable to attach C-terminal additions longer than 30-residues (e.g., RBM72ext, Figure 1C) to the capsid, presumably due to similar inter-subunit clashes. The incorporation of flexible linkers between the core capsid shell and the displayed components may alleviate some of these limitations.

The DNA vaccine component as represented in phage DEaDAS warrants further investigation. The expressed RBD protein did not elicit an immune response greater than seen in the parental BaDAS-1 phage, but this could also result from conformational limitations. Nonetheless, the genome capacity of the phage is anticipated to accommodate 3–4 kb of additional DNA, enabling a variety of recombinants to be constructed that may overcome these constraints. These could be combined with alternative virion modifications including decoration with peptides that promote uptake of the particles by antigen-presenting cells.

A potential benefit of these mycobacteriophages is that the C-terminal domains on the capsid and tail tube proteins are highly immunogenic and dominate the immune response to the phages (Figure 5). It is plausible that these domains act as immune decoys and distract the immune system from responding to either the displayed peptides or to other phage components such as receptor binding proteins at the tail tip, giving only mild neutralization (Figure 4B). However, we note that BaDAS-5 did not elicit greater S-specific responses than BaDAS-1, and thus the tail tube extension does not obviously act as in this way. Nonetheless, these observations suggest that if larger and properly folded protein segments can be successfully displayed (such as the whole RBD)—perhaps through the use of peptide linkers—they could be highly immunogenic and might elicit neutralizing SARS-CoV-2 antibody responses.

There are several potential limitations to mycobacteriophage-based vaccine technology. First, antigen size is a potential constraint, and it is unlikely that large protein segments can be added to either the capsid or tail tube. For example, we were not successful in adding a 72-residue RBM segment, although exploration of various peptide linkers may help to overcome this. Furthermore, interactions between covalently bound antigens could also limit construction by preventing successful self-assembly of phage particles. Secondly, genome capacity is an important consideration, because genome length must be within the packaging limits of the virion. This is especially relevant to the DNA vaccine facet, where multi-gene expression cassettes and long antigen sequences could span several kbp. Genome length constraints can likely be mitigated by deleting non-essential genes to make space for vector and antigen sequences. Other phage-based vaccines platforms avoid some of these limitations [17]. Finally, for successful constructions there remains the question of how the immune system reacts to phage-based vaccination, and whether peptides are displayed in conformationally suitable forms that promote neutralizing immune responses.

## Figures and Tables

**Table 1:** Recombinant phages constructed in this study.

Recombinant Phage	Description of Mutations
phiFW1	gp14 truncated after D304
phiFW2	gp14 truncated after D304 gp19 truncated after V213
BaDAS-1	RBM30 inserted after gp14 S395
BaDAS-3	RBM30 inserted after gp14 G397
BaDAS-4	Nterm RBM30 inserted after gp14 G397
BaDAS-5	RBM30 inserted after gp14 S395 gp19 truncated after V213
BaDAS-6	RBM30 inserted after gp14 G397 gp19 truncated after V213
BaDAS-7	Nterm RBM30 inserted after gp14 G397 gp19 truncated after V213
DEaDAS-1	RBM30 inserted after gp14 S395 RBD expression cassette substituted for phiTM45 integrase, gene *35*

**Table 2:** Studies of mice inoculations with vaccine candidates.

Study	Group	# of Mice/Group	Age of Mice (Weeks)	Sex of Mice	Prime	Prime Dose (pfu or µg)	Prime Adjuvant	Boost	Boost Dose(pfu or µg)	Boost Adjuvant	Bleed & Boost Schedule (Weeks Post Prime)
1	1	4	15–20	F M	PBS	N/A	N/A	PBS	N/A	N/A	2—bleed 2—boost 4—bleed 4—boost 6—bleed 8—bleed
	2	4	15–20	F M	phiTM45	1E10	N/A	phiTM45	1E10	N/A	
	3	4	15–20	F M	phiTM45	1E11	N/A	phiTM45	1E11	N/A	
	4	4	15–20	F M	BaDAS-1	1E10	N/A	BaDAS-1	1E10	N/A	
	5	4	15–20	F M	BaDAS-1	1E10	5 µg LPS	BaDAS-1	1E10	5 µg LPS	
	6	4	15–20	F M	BaDAS-1	1E11	N/A	BaDAS-1	1E10	N/A	
2	1	5	8	F	PBS	N/A	N/A	PBS	N/A	N/A	3—bleed 3—boost 6—bleed 9—bleed 12—bleed
	2	5	8	F	Spike S1	20	5 µg LPS	Spike S1	20	5 µg LPS	
	3	5	8	F	phiTM45	1E11	N/A	phiTM45	1E11	N/A	
	4	5	8	F	phiTM45	1E11	5 µg LPS	phiTM45	1E11	5 µg LPS	
	5	5	8	F	BaDAS-1	1E11	N/A	BaDAS-1	1E11	N/A	
	6	5	8	F	BaDAS-1	1E11	5 µg LPS	BaDAS-1	1E11	5 µg LPS	
	7	5	8	F	BaDAS-3	1E11	N/A	BaDAS-3	1E11	N/A	
	8	5	8	F	BaDAS-3	1E11	5 µg LPS	BaDAS-3	1E11	5 µg LPS	
	9	5	8	F	BADAS-4	1E11	N/A	BADAS-4	1E11	N/A	
	10	5	8	F	BaDAS-4	1E11	5 µg LPS	BaDAS-4	1E11	5 µg LPS	
3	1	3	12	F M	BADAS-5	5E10	N/A	N/A	N/A	N/A	2—bleed 4—bleed
	2	3	12	F M	BaDAS-6	5E10	N/A	N/A	N/A	N/A	
	3	3	12	F M	BaDAS-7	5E10	N/A	N/A	N/A	N/A	
4	1	3	12	F	BaDAS-1	5E10	N/A	BaDAS-1	5E10	N/A	3—bleed 3—boost 6—bleed 9—bleed
	2	3	12	F	BaDAS-1	5E10	N/A	Spike S1	10	N/A	
	3	3	12	F	BaDAS-5	5E10	N/A	BaDAS-5	5E10	N/A	
	4	3	12	F	BaDAS-5	5E10	N/A	Spike S1	10	N/A	
5	1	3	8	F M	RBD	2	2.5 µg LPS	RBD	2	2.5 µg LPS	3—bleed 3—boost 6—bleed 9—bleed
6	1	3	12	F	DEaDAS-1	5E9	N/A	DEaDAS-1	1E11	N/A	3—bleed 6—bleed 11—boost 14—bleed
7	1	4	8	F	PBS	N/A	N/A	Spike S1	10	N/A	3—bleed 6—boost 9—bleed
	2	4	8	F	BaDAS-1	1E11	N/A	BaDAS-1 + Spike S1	1E11 + 10	N/A	
	3	4	8	F	DEaDAS-1	1E11	N/A	Spike S1	10	N/A	

## Data Availability

The data presented in this study are available within the article and supplementary materials.

## Supplementary Materials

The following are available online at https://www.mdpi.com/article/10.3390/microorganisms9122414/s1, Figure S1. Additional ELISA endpoint titer summaries, Figure S2. Western blot showing reactivity of BaDAS-1 sera to SARS-CoV-2 RBD, Figure S3. Western blot further supporting immunogenic independence of capsid and tail tube extensions, Figure S4. Additional FRNT results show no neutralization.
