# Supplementary material for: A Mycobacteriophage-Based Vaccine Platform: SARS-CoV-2 Antigen Expression and Display
Source: Microorganisms. 2021 Nov 23;9(12):2414. doi: 10.3390/microorganisms9122414 (PMC8704799; doi:10.3390/microorganisms9122414)
Supplement: Supplementary file 1 [file microorganisms-09-02414-s001.zip › microorganisms-1459989-supplementary.pdf]

Supplementary Materials

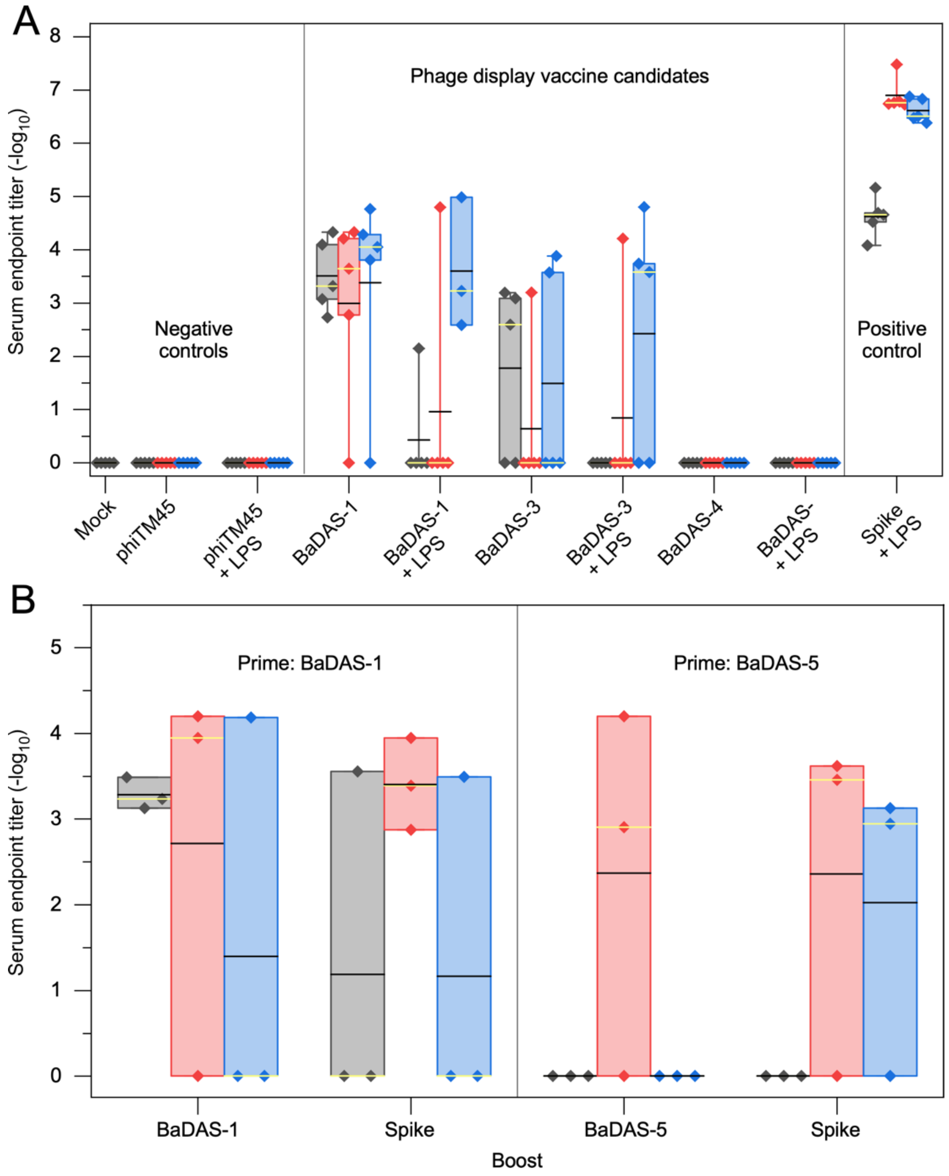

Figure S1. Additional ELISA endpoint titer summaries. Reciprocal serum endpoint titers of all inoculated animals in Studies 2 (A) and 4 (B). Titers for individual animals are shown as diamond symbols (with whiskers extending to the minima and maxima of each dataset) and box boundaries mark the first and third quartiles of the data. Black and yellow lines are the mean and median endpoint titer, respectively. Shaded to indicate responses 3 weeks (gray), 6 weeks (red) or 9 weeks (blue) after primary inoculation.

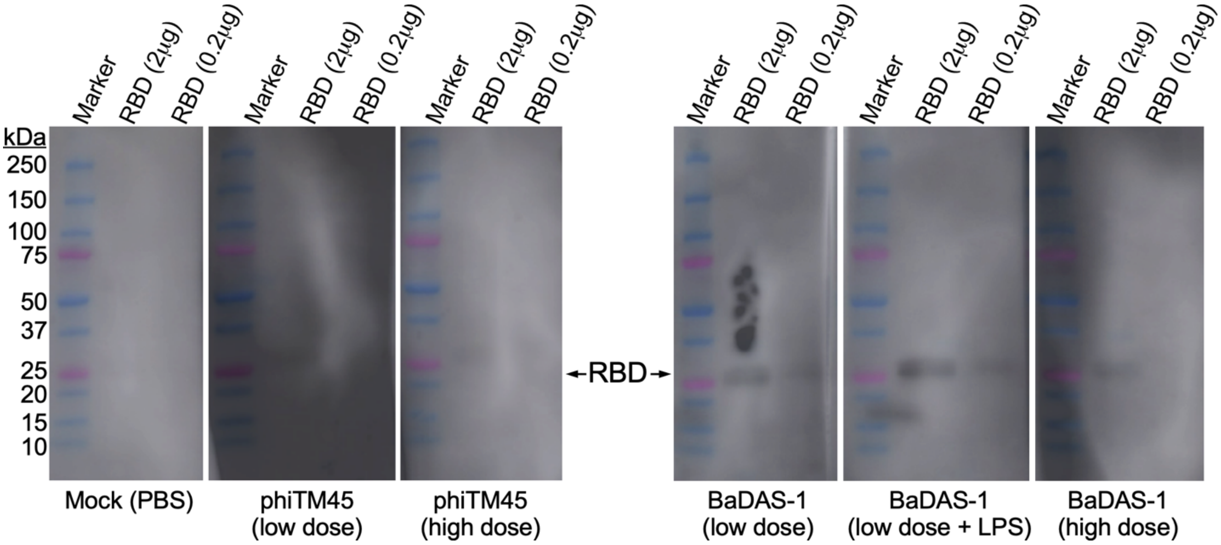

Figure S2. Western blot showing reactivity of BaDAS-1 sera to SARS-CoV-2 RBD. Purified RBD (2µg and 0.2µg) was separated on 4-20% SDS PAGE gels, then transferred to PVDF membranes, cut into strips, and probed with sera as indicated below each blot. While very faint reactivity is observed to phiTM45-immunized mice, much stronger reactivity is observed in the three sera from animals immunized with BaDAS-1.

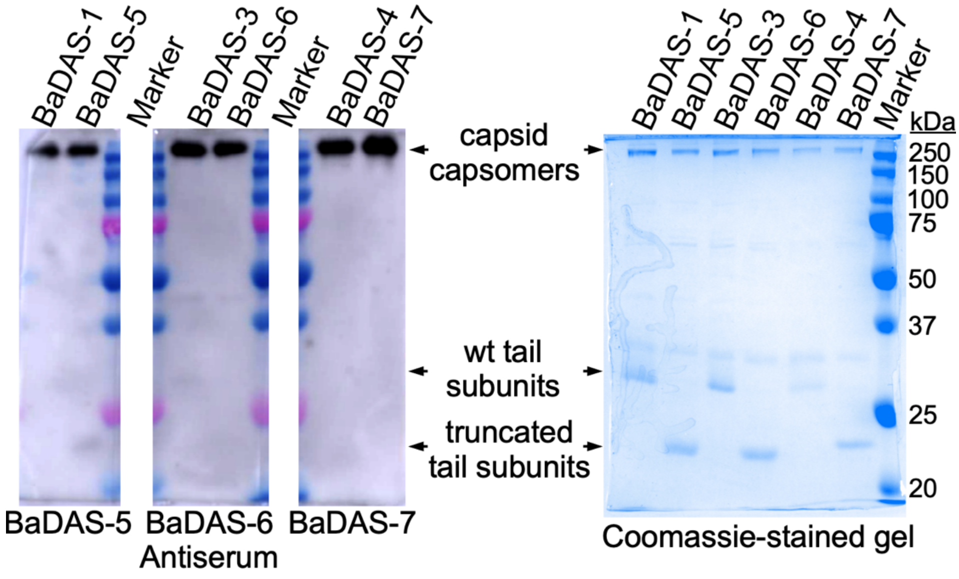

Figure S3. Western blot further supporting immunogenic independence of capsid and tail tube extensions. As shown in Figure 6, immunization with BaDAS-5, -6, and -7 (which have truncated tail tube extensions) produces serum that reacts only to capsid proteins. This illustrates that capsid-binding antibodies do not cross-react with the natural C-terminal extension on the tail tube protein. It also confirms that the natural C-terminal extension is the immunodominant component of the tail tube; when the extension is removed, no reactivity to the remaining tail tube is observed.

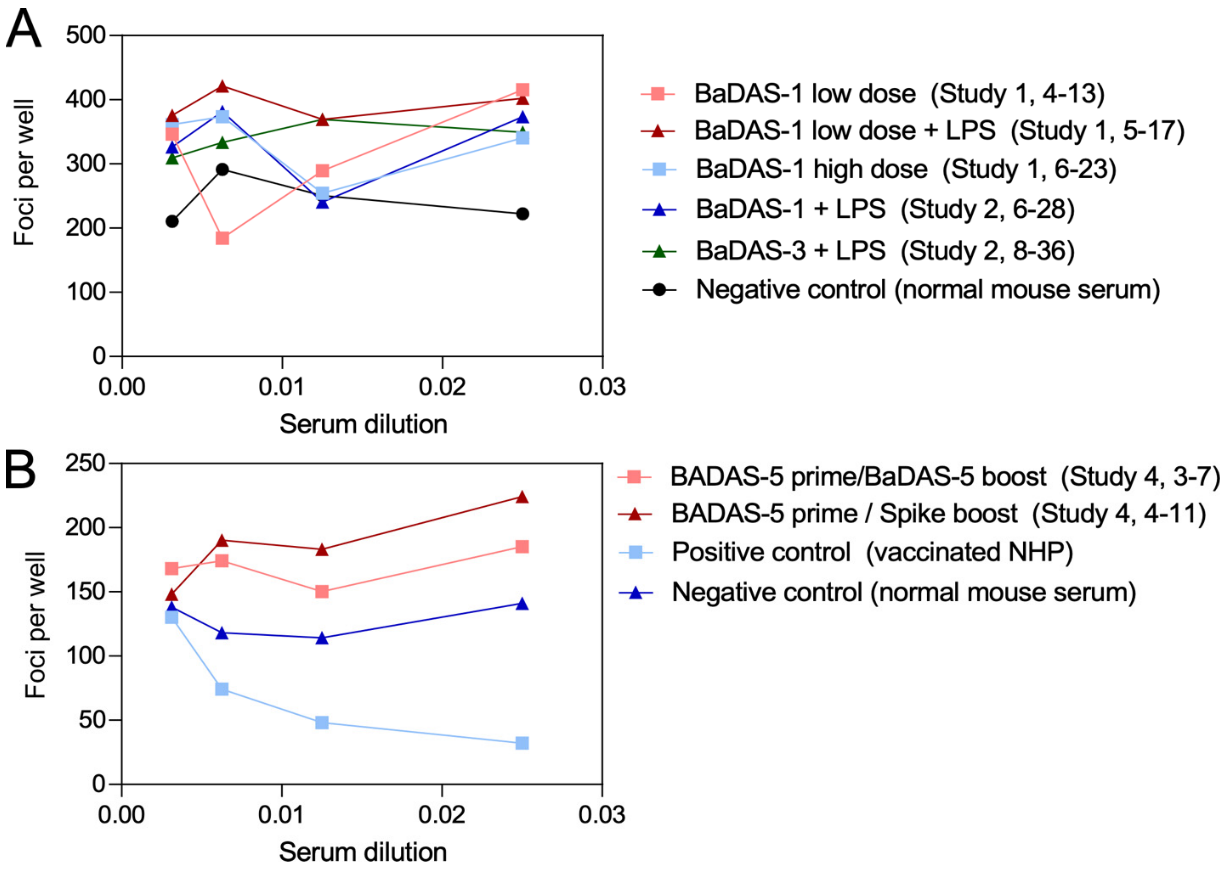

Figure S4. Additional FRNT results show no neutralization. As in Figure 7, no reduction in the number of foci per well is observed in serum from mice immunized with BaDAS-1, even when varying the dosage or using adjuvant. Furthermore, BaDAS-3 and -5 are also not effective in producing neutralizing antibodies, even with adjuvant or heterologous prime / boost strategies.
